# A machine-vision framework for electric vehicle charging hole localization based on contour-driven detection and geometric verification

**DOI:** 10.1371/journal.pone.0359570

**Published:** 2026-10-22

**Authors:** Wei Luo, Qiantao Huo, Xin Li

**Affiliations:** 1 Nanjing University of Science and Technology Zijin College, Nanjing, China; 2 NARI Technology Development Co. Ltd, Nanjing, China; 3 State Grid Electric Power Research Institute, Nanjing, China; Southeast University, CHINA

## Abstract

The accurate localization of charging holes is an indispensable perception step for the automatic charging system of electric vehicles, since the success of subsequent charging-gun insertion depends directly on the precise estimation of the geometric centers of the holes on the vehicle charging interface. This paper presents a lightweight machine-vision framework for charging hole localization based on contour-driven detection and geometric verification. The proposed framework integrates image filtering, RGB-to-HSL color-space conversion, parameterized HSL adjustment, two-stage contour detection, minimum enclosing circle fitting, and Hough circle verification into a deterministic pipeline. The framework is interpretable, does not require a learning-based training process, and can be deployed on conventional CPU-based computing resources. The image data used in this study were acquired by a monocular RGB camera mounted on the charging robot end-effector, in the same sensing configuration in which the system is intended to operate. A total of 80 images were collected from six vehicles equipped with the same five-hole charging interface, under realistic variations of illumination, viewing angle, reflective interference, and background complexity. Detailed parameter selection rationale, a parameter perturbation analysis, and a condition-specific ablation analysis are reported. On an Intel Core i7-12700 CPU with 16 GB RAM and no GPU acceleration, the proposed framework achieves an overall mean localization error of 4.2 pixels and a 96.25% detection success rate, with an average per-image processing time of approximately 60 ms. The results demonstrate that the proposed framework provides accurate, robust, and engineering-deployable charging-hole localization for the target charging interface.

## 1. Introduction

With the popularization and promotion of electric vehicles [1–3], automatic charging systems for electric vehicles have a wide range of needs and application prospects. After receiving a charging command, the electric vehicle automatic charging system can select the nearest idle charging pile, move the charging gun along the path, search for and locate the vehicle charging seat, and achieve automatic charging; after charging is completed, the charging gun can be put back in place and the next charging command can be waited for or executed. The electric vehicle automatic charging system is the technical guarantee for unattended charging parking lots. Many studies on automatic charging systems [4–7] have been published.

An essential part of the automatic charging system for electric vehicles is the sensing and positioning of the charging holes [8]. After completing this function, the automatic connection between the charging gun and the electric vehicle can be achieved. There are various methods for studying the positioning of charging holes [9–11], including some based on machine vision technology [12–14]. However, most of these documents focus on principles and rarely involve experimental processes and parameter processing. In addition, recent research has explored other complementary aspects of electric vehicle charging infrastructure, including resilient charging demand forecasting under cyberattacks [15,16] and curriculum-guided multi-feature fusion transformer-based charging monitoring systems [17]. These works share with the present study the broader goal of improving the intelligence and operational reliability of EV charging systems, but they target a different layer of the system (forecasting and system-level monitoring) rather than the precise vision-based localization of the charging hole that is the focus of this paper. Based on machine vision technology, an automatic identification method for charging holes to determine the specific location of charging holes in three-dimensional space is proposed in this paper. The overall implementation process, as well as the experimental process and results of each step, is detailed instead of an introduction to the principle. This method breaks through key technologies and has detailed data, and it has been implemented in the automatic charging system studied by the author [4].

The contributions of this paper are as follows. (i) We present a deterministic, lightweight, and interpretable contour-driven machine-vision framework for charging hole localization that can be deployed on conventional CPU hardware. (ii) We provide a transparent description of the dataset and the image-acquisition setup, including the camera configuration on the charging robot end-effector and the realistic operating variations represented in the data. (iii) We provide a comprehensive parameter table and a parameter perturbation analysis, so that the engineering rationale and stability of the framework can be reproduced and assessed. (iv) We provide a condition-specific ablation analysis that links each component of the framework to the type of image difficulty it is designed to mitigate. (v) We explicitly delineate the scope of the present implementation: the framework is validated on one charging interface type with five circular holes, and the generic part of the pipeline is distinguished from the interface-specific geometric prior used in the verification stage.

## 2. System architecture and workflow

The automatic charging system mentioned in this paper is divided into three subsystems: the charging hole positioning subsystem, the automatic charging execution subsystem, and the communication/control subsystem. The relationship between the three subsystems is shown in Fig 1. The charging hole positioning subsystem is divided into two parts: a regional positioning module and a precise positioning module. The system works by first performing area positioning/overall movement, followed by precise positioning/control. Regional positioning realizes the function of positioning the charging area of the electric vehicle, which is completed by the regional positioning module shown in Fig 1; precise positioning, which realizes the function of positioning the charging hole of the electric vehicle, is completed by the precise positioning module which is the key issue of this paper, also shown in Fig 1.

**Fig 1:**
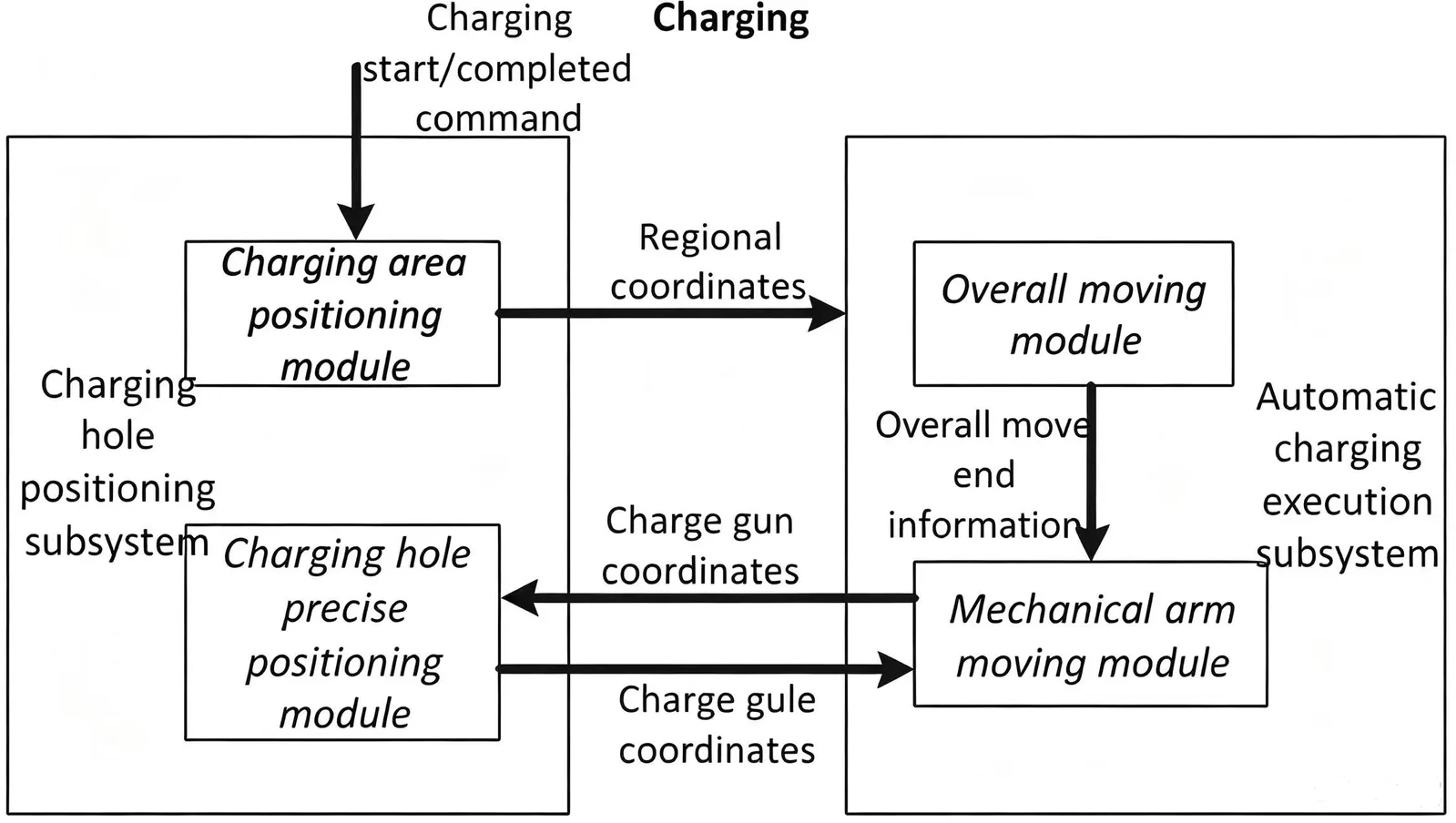
Overall framework diagram of the automatic charging system.

In this work, the perception module is integrated with the charging robot rather than separated as a fixed external observer. The image of the charging interface is acquired by a monocular RGB camera that is mounted on the charging robot end-effector. The camera resolution is 1920 × 1080 pixels, and its intrinsic parameters were calibrated in advance. The motivation for adopting an end-effector-mounted camera is that this configuration is consistent with the practical operating geometry of the automatic charging system: the camera observes the charging port from the manipulator side, and the perception result can be directly used to guide the subsequent motion of the charging gun. As a consequence of this configuration, the imaging geometry between the camera and the charging port is controlled but not perfectly identical across all images, since the end-effector pose, illumination conditions, reflectance, and surrounding background can all vary in practice.

The workflow of the overall system is therefore organized as a sequence of stages: (1) coarse regional positioning, in which the charging robot is moved into an appropriate observation region relative to the charging interface; (2) image acquisition, in which the end-effector-mounted camera captures an image of the charging port at a designated observation pose; (3) precise localization, in which the proposed machine-vision framework is applied to the captured image to estimate the geometric centers of the charging holes; and (4) motion execution, in which the estimated centers are used by the control module to guide the charging gun toward final alignment and insertion. The proposed method addresses stage (3). It is therefore performed on images acquired at a stable observation pose after coarse positioning, rather than as a continuously running visual search module during arbitrary end-effector motion. This distinguishes the present work from full visual servoing during continuous motion: here, perception and motion are sequential rather than concurrent.

## 3. Charging hole identification method

This function belongs to the precise positioning module in the charging-pole positioning subsystem. After filtering, transforming color space, traversing HSL space, contour detection, drawing the smallest circle, and Hough circle detection (six processes), the charging hole is finally identified. The flowchart is shown in Fig 2.

**Fig 2:**
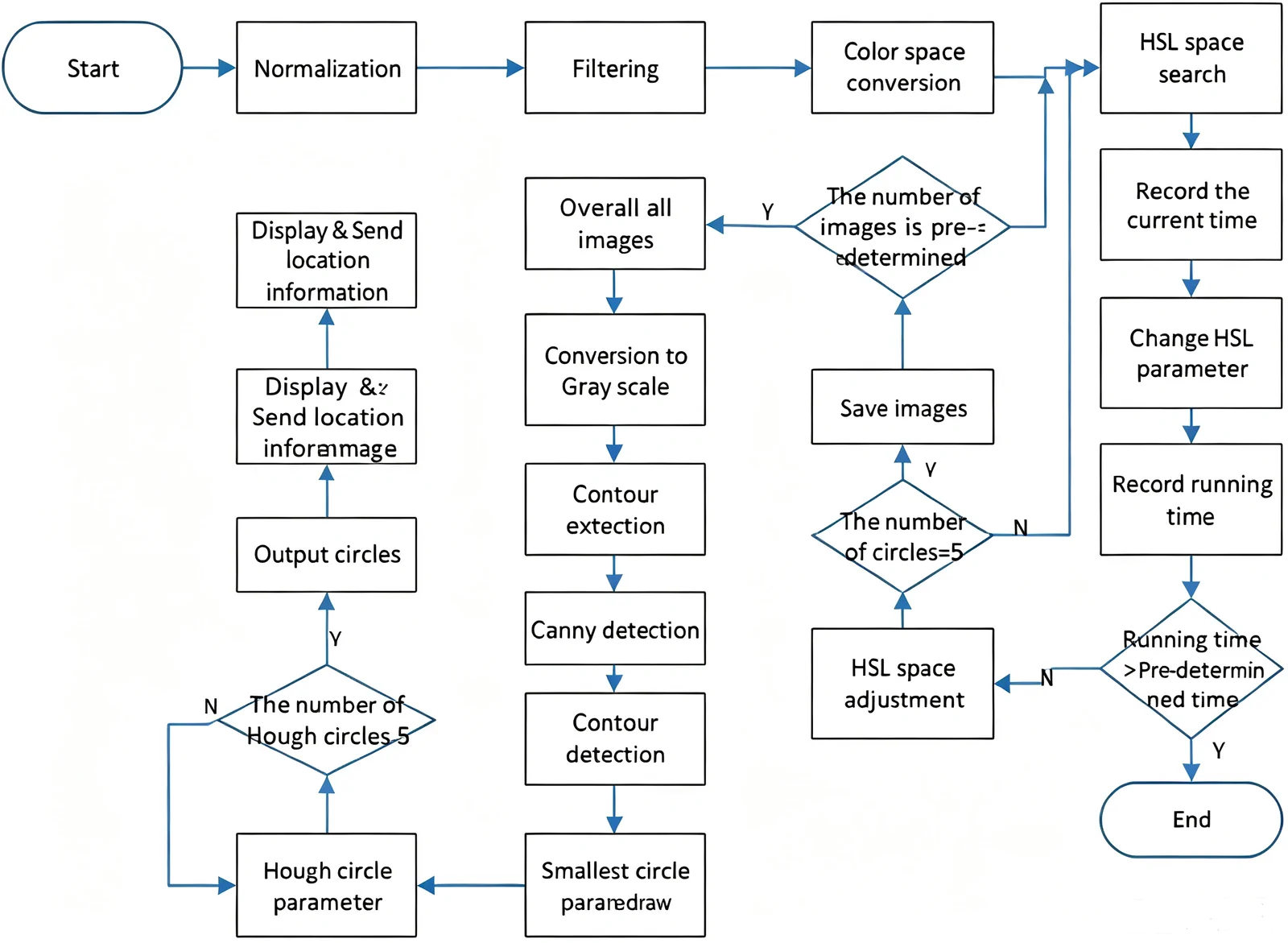
Charging hole identification flowchart.

It is worth emphasizing that the proposed framework is rule-based and deterministic. It does not contain random weight initialization, iterative training, or data-driven parameter fitting. For a fixed input image and a fixed parameter configuration, the algorithm always produces the same output. The Hough verification stage uses an automatic feedback loop that adjusts a small number of detection parameters until the geometric prior of the target charging interface is satisfied (i.e., the expected number of circles is detected); this loop is itself deterministic for a given image and a given starting parameter configuration. As a result, the validation protocol of this method differs from that of trainable models: the relevant question is not stability across stochastic re-runs, but stability with respect to the chosen parameter configuration, which is analyzed in Section 5.2.

## 4. Experimental dataset and implementation environment

### 4.1. Dataset

The proposed framework was evaluated on a self-collected dataset acquired on a real electric vehicle automatic charging platform. The dataset contains 80 images acquired from 6 distinct electric vehicles. All images correspond to the same charging port type, namely a five-hole charging interface, which is the target interface configuration of the automatic charging system studied in this work. The dataset is therefore interface-specific by design: it is intended to validate the proposed framework on one practical charging interface rather than across heterogeneous charging standards.

Although the charging-port type is fixed, the dataset deliberately contains realistic variability at the image level, reflecting the conditions under which the automatic charging system is expected to operate. Specifically, the 80 images cover the following four practical conditions: (i) normal illumination, (ii) low-light conditions, (iii) reflective surfaces or visually ambiguous geometric features (for example, when the metallic charging-port surface produces highlights that visually compete with the holes), and (iv) mild viewpoint variation around the nominal observation pose. These four conditions are also used as the grouping factor in the condition-specific ablation analysis reported in Section 6. Representative samples illustrating each of these conditions are shown in Fig 3.

**Fig 3:**
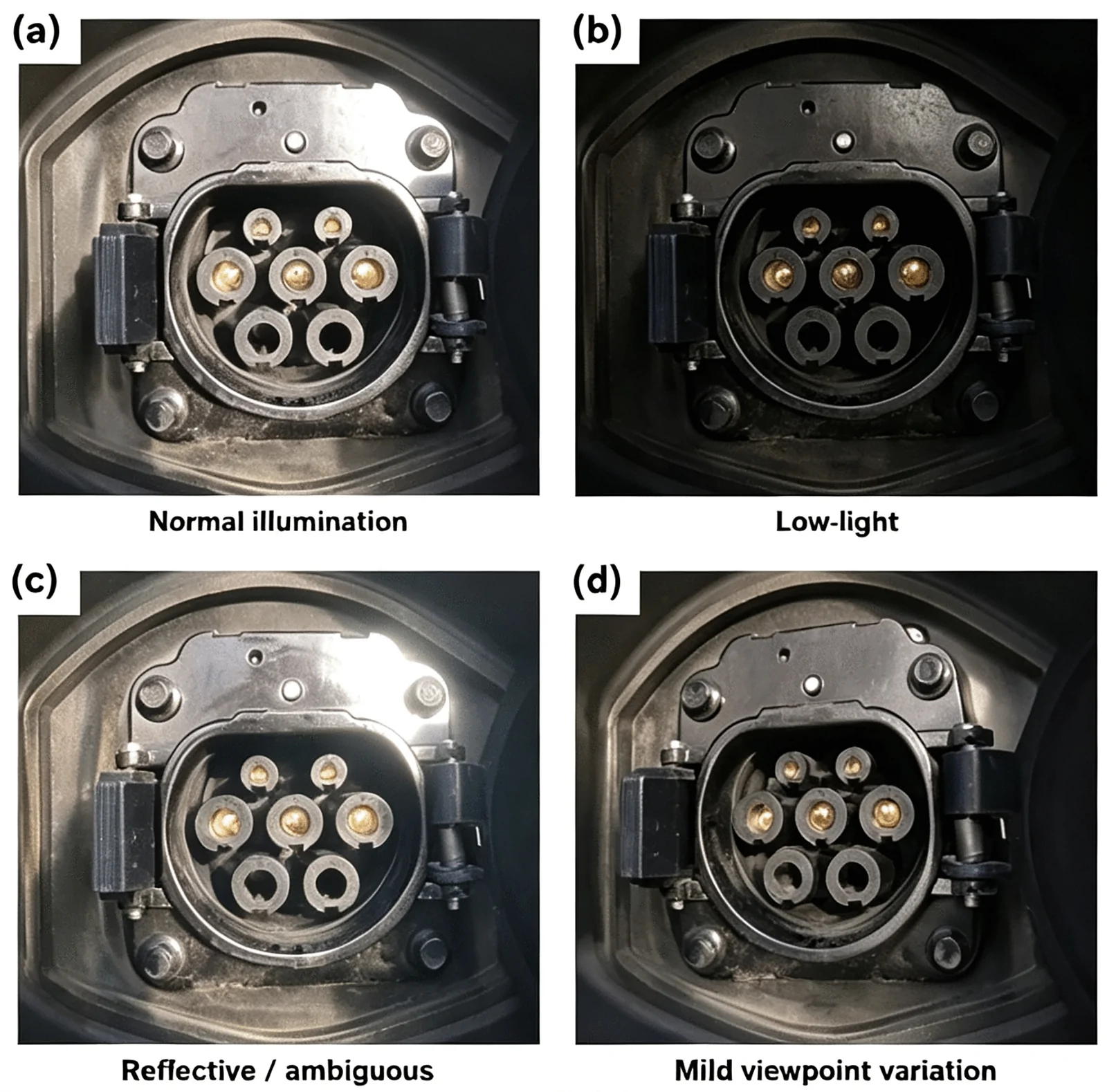
Representative samples from the self-collected dataset under different practical conditions.

All images were acquired in the same sensing configuration described in Section 2, namely a monocular RGB camera mounted on the charging robot end-effector with a resolution of 1920 × 1080 pixels. During acquisition, the camera-to-charging-port geometry was controlled to match the practical operating configuration of the automatic charging system, so the images do not represent unconstrained in-the-wild capture. However, the acquisition conditions are not identical across all images: the dataset includes moderate but realistic variation in illumination level, viewing angle, reflective interference, and background complexity. This makes the dataset more representative of practical operating conditions than a single repeated capture would be, while still maintaining a sufficiently stable geometric relationship for precise localization.

### 4.2. Implementation environment

The proposed framework is implemented in Python using the OpenCV computer vision library. All experiments reported in this paper, including localization accuracy, parameter perturbation, condition-specific ablation, and runtime measurements, were performed on the following hardware and software environment:

• CPU: Intel Core i7-12700. • RAM: 16 GB. • Operating system: Windows 10, 64-bit. • Software: Python with OpenCV. • GPU acceleration: not used.

This configuration was deliberately chosen to be representative of conventional CPU-based computing resources rather than specialized high-end GPU hardware, since the proposed framework is intended as a lightweight and engineering-deployable solution. As reported in Section 6.4, the proposed framework processes each image in approximately 60 ms on this platform; this number reflects the algorithmic pipeline (preprocessing, HSL conversion and adjustment, contour-based detection, geometric fitting, and Hough verification) and does not include image acquisition from the camera, external communication, or the mechanical motion time of the charging robot. We make this distinction explicit so that the reported runtime is not confused with the overall execution time of the full charging system.

## 5. Method implementation and step-by-step analysis

### 5.1. Filtering

The main purpose of filtering is to reduce noise in the image and minimize errors caused by noise interference. In this paper, three filtering methods, mean blur, Gaussian blur, and median filter, are tested. In fuzzy processing, there is a key variable named convolution kernel that needs to be adjusted, and using different convolution kernel parameters yields different results. Two convolution kernel sizes, 3 × 3 and 5 × 5, are tested in this paper. The experimental results are shown in Fig 4. Based on the table, we can compare the differences between the three filtering methods and the impact of using different convolution kernels on the image under the same filtering method. The corresponding default values and tuning ranges are summarized in Table 1 (Section 5.7).

**Table 1:** Key parameter settings of the proposed charging hole localization framework.

Stage	Parameter	Default	Tuning range	Selection rationale
Filtering	Filter type	Gaussian	Mean/ Gaussian/ Median	Suppress noise while preserving circular contour structure.
Filtering	Convolution kernel	3 × 3	3 × 3/ 5 × 5	Balance smoothing strength against blurring of small charging-hole features.
HSL adjustment	Lightness factor	1.20	1.10–1.30	Improve separability of dark charging holes from background under varying illumination.
HSL adjustment	Saturation factor/ Hue offset	1.10/ 0°	0.90–1.20/ −5°–5°	Enhance contrast between charging-hole region and surrounding metallic surface while avoiding excessive color distortion.
Quick contour detection	Contour line thickness	2 px	1–3 px	Ensure connected contours of candidate hole regions.
Quick contour detection	Drawn-circle line thickness	12 px	8–16 px	Fill the candidate hole region without spilling into adjacent regions.
Min. enclosing circle	Initial smallest-circle radius	R/2	0.4R–0.6R	Provide a stable seed radius relative to the known charging-hole physical size.
Min. enclosing circle	Min.-circle line thickness	R	0.8R–1.2R	Eliminate residual gaps inside the candidate region for stable subsequent verification.
Hough verification	Accumulator threshold	18	16–20	Balance true-circle detection rate against false circles in cluttered regions.
Hough verification	Min/ Max circle radius	22/ 45 px	18–50 px	Constrain detection to the physically plausible charging-hole radius range in the captured images.
Hough verification	Expected number of holes	5	Interface-specific	Geometric prior of the target five-hole charging interface (see Section 8).

**Fig 4:**
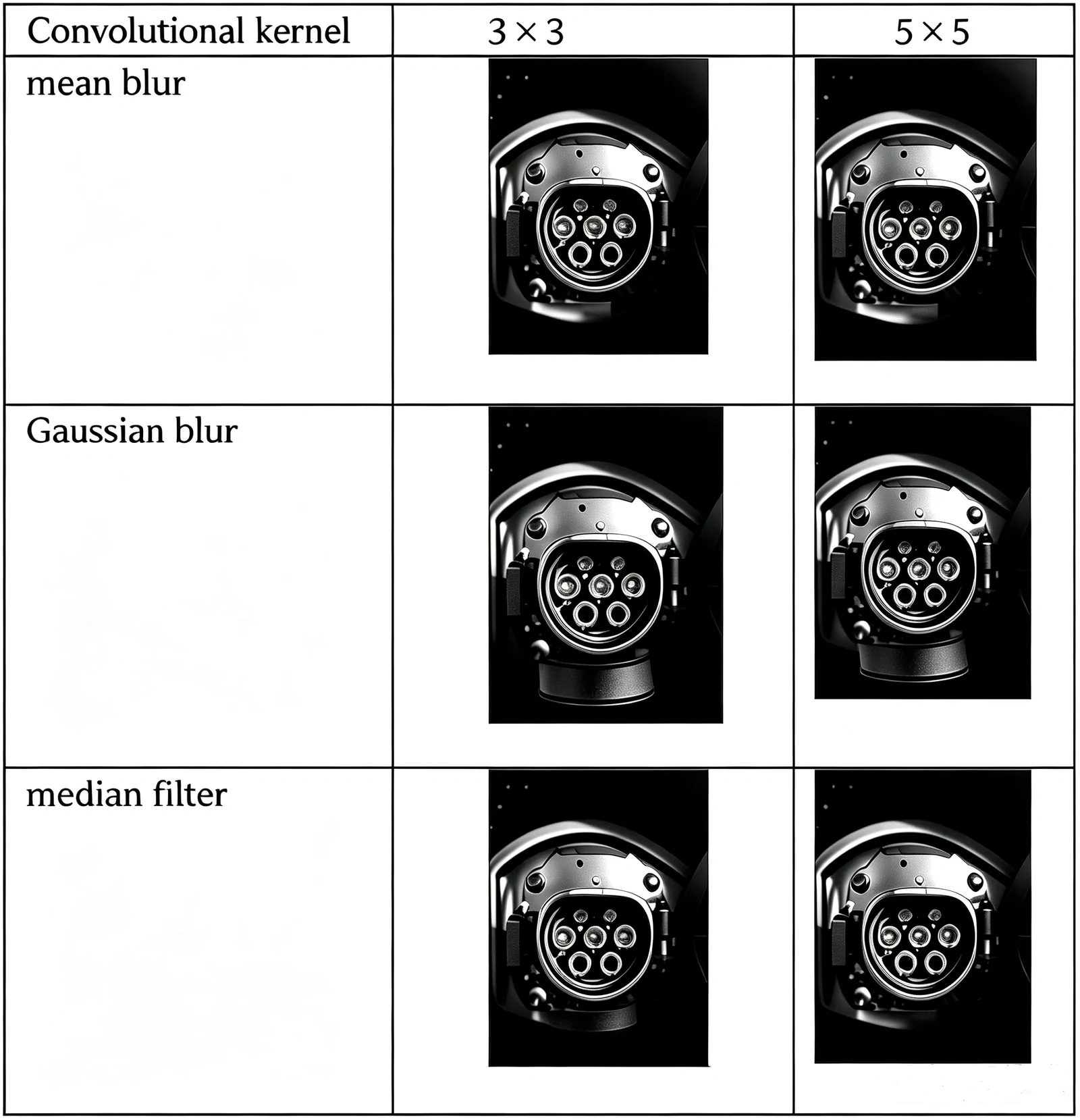
Comparison of different filtering methods.

### 5.2. Color space conversion

After filtering is completed, the conversion from RGB color space to HSL space is performed. The converted image is shown in Fig 5 and serves as input to the subsequent modules.

**Fig 5:**
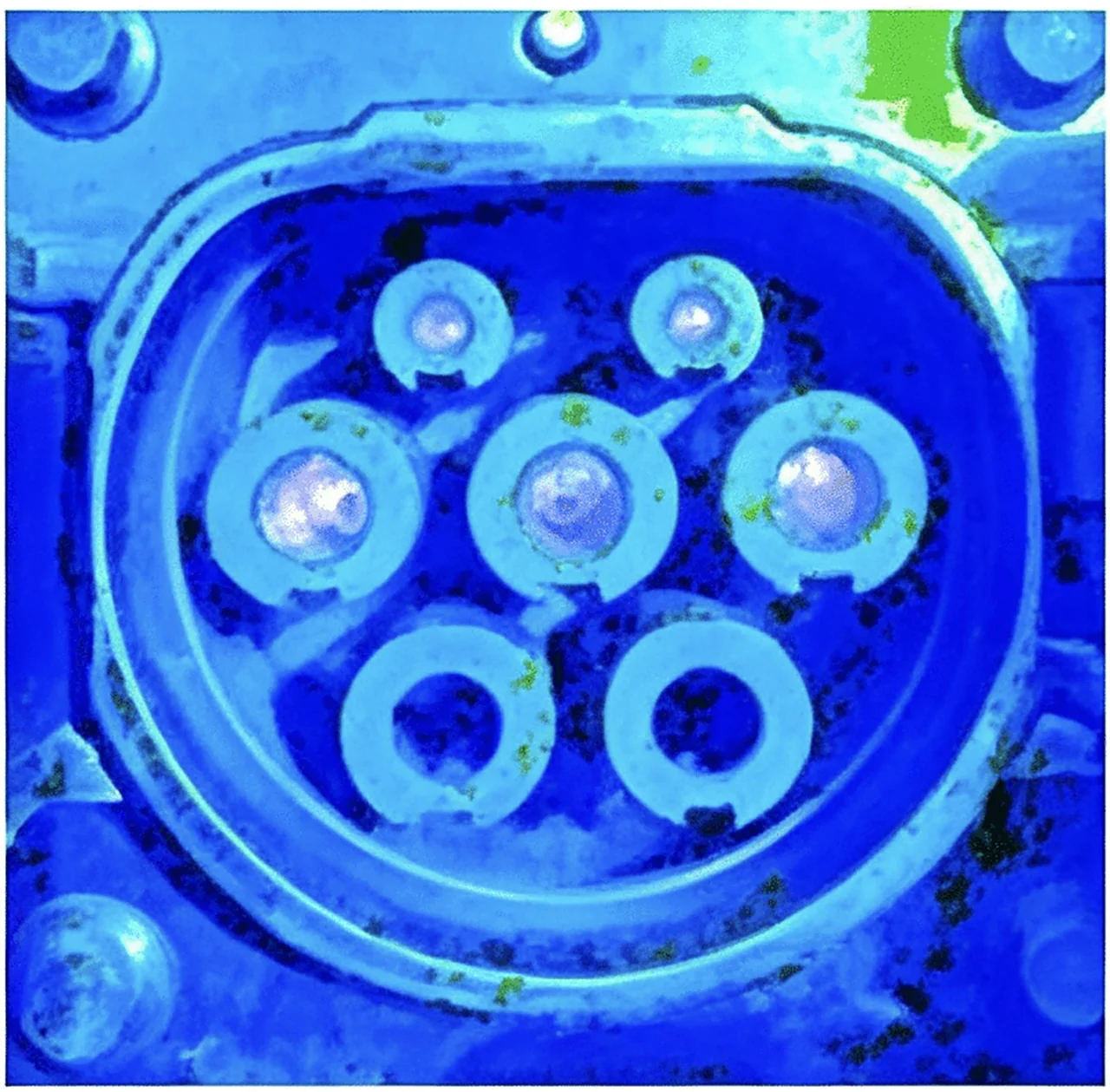
HSL image.

### 5.3. HSL space adjustment

Different H, S, and L values are used for function transformation. After the transformation is completed, the transformed output image is saved, as shown in Fig 6. Output images are different with different parameters, which may have an impact on subsequent processing, and may even influence whether the charging hole position can be accurately identified.

**Fig 6:**
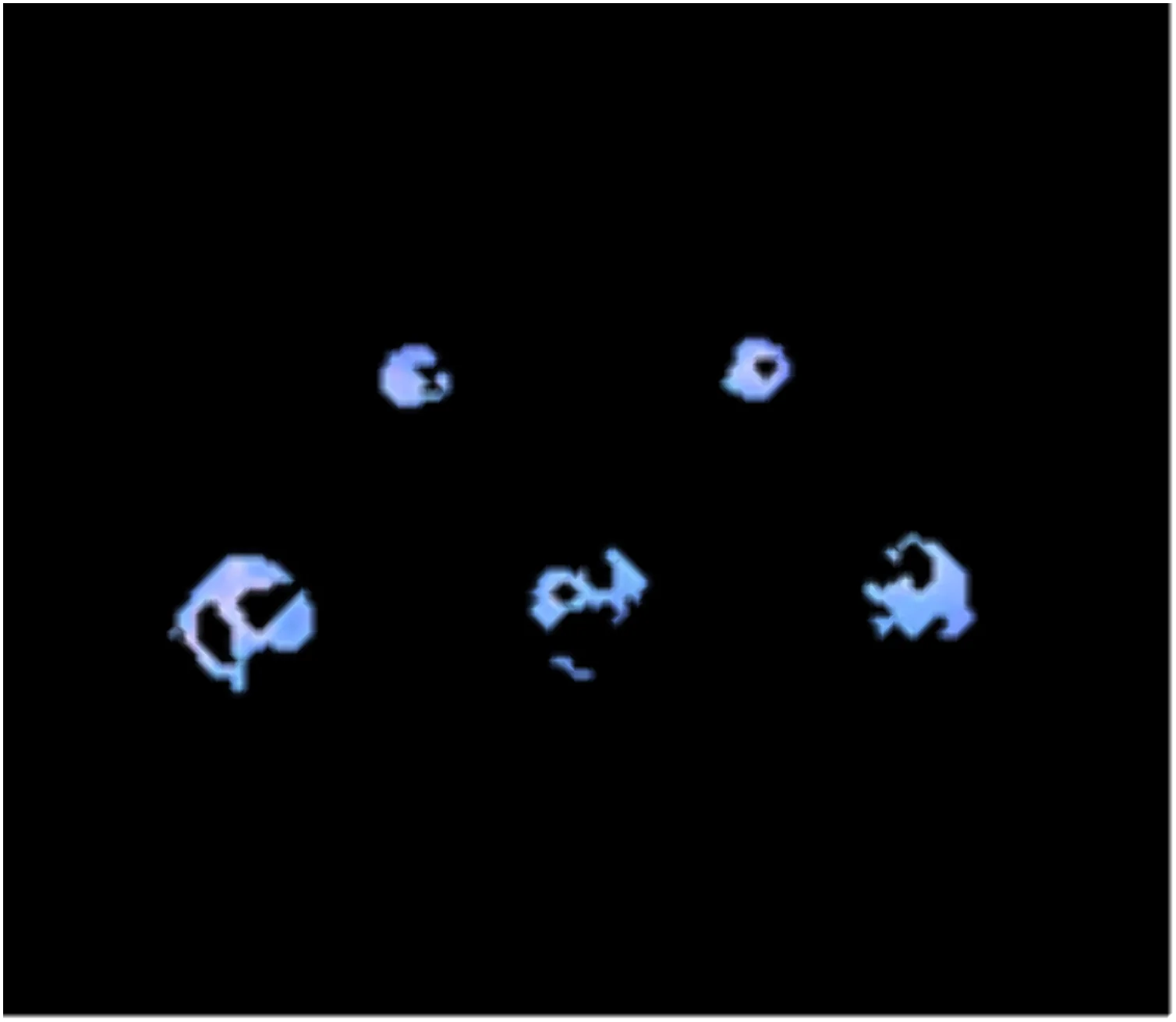
Image after HSL spatial transformation.

### 5.4. Contour detection

#### 5.4.1. Quick detection.

First, edge detection is performed on the image to obtain an image that contains only edges. Then, contour search is performed, and the approximate contour and the smallest enclosing circle are drawn. Appropriate parameters are needed to fill the middle area of the charging hole as much as possible. The parameters for drawing the smallest circle should also ensure that the detected smallest circle is fully filled; otherwise, it will affect subsequent recognition and judgment. The parameters selected for this experiment are: contour line thickness of 2 px, drawn-circle line thickness of 12 px, initial smallest-circle radius of R/2, and smallest-circle line thickness of R.

After that, the image is converted to grayscale for subsequent operations. Then contour search is performed again. This time, because the inside of the circle has been filled, it only needs to detect whether the number of contours is five (the number of charging holes). If the detection result is five, the condition is met; otherwise, the condition is not met and the procedure is repeated. This corresponds to actually detecting five regions that are expected to correspond to the five charging holes. Note that the requirement that exactly five contours be detected is an interface-specific geometric prior of the target five-hole charging interface; the implications of this prior for generalizing the framework to other charging standards are discussed in Section 8.

Fig 7 shows the detection result of an image in this module. The contour is significantly larger than the actual size; this is because this detection is only for the purpose of quickly obtaining usable images, without the need for fine rendering. The precise location is achieved in the next step.

**Fig 7:**
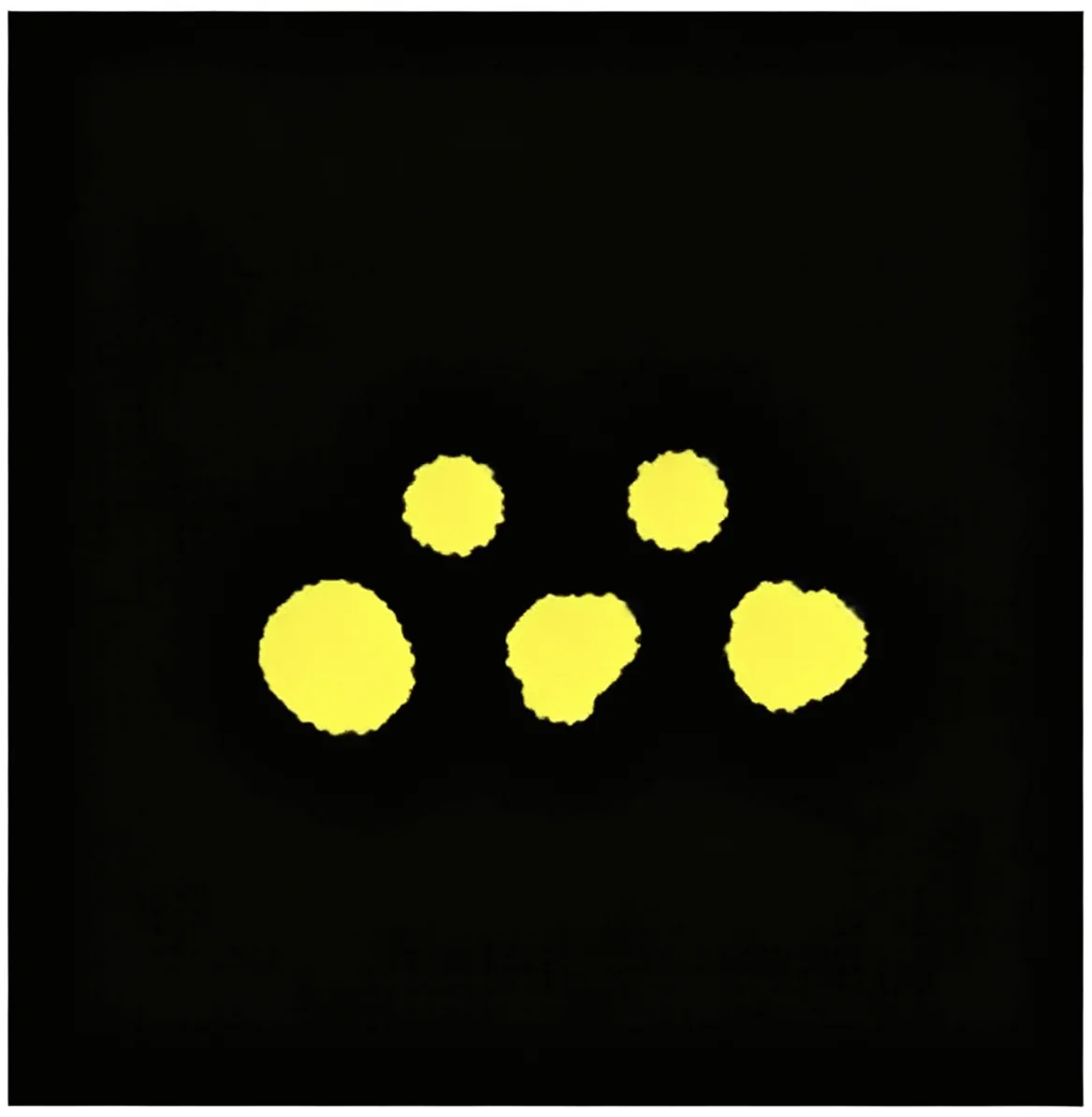
Quick detection result.

#### 5.4.2. Accurate detection.

The input to this module is the quickly detected image. After searching for contours and drawing them using thicker lines, the output is five dense regions without gaps. Then, edge detection is performed on this region to obtain five accurate contour maps.

Fig 8(a) and 8(b) show the image and outline after the second detection. Because the final valid data to be obtained are the five circle centers, independent of the radius, the thickness setting of the line does not have a significant impact in this step, as long as the five contours do not overlap.

**Fig 8:**
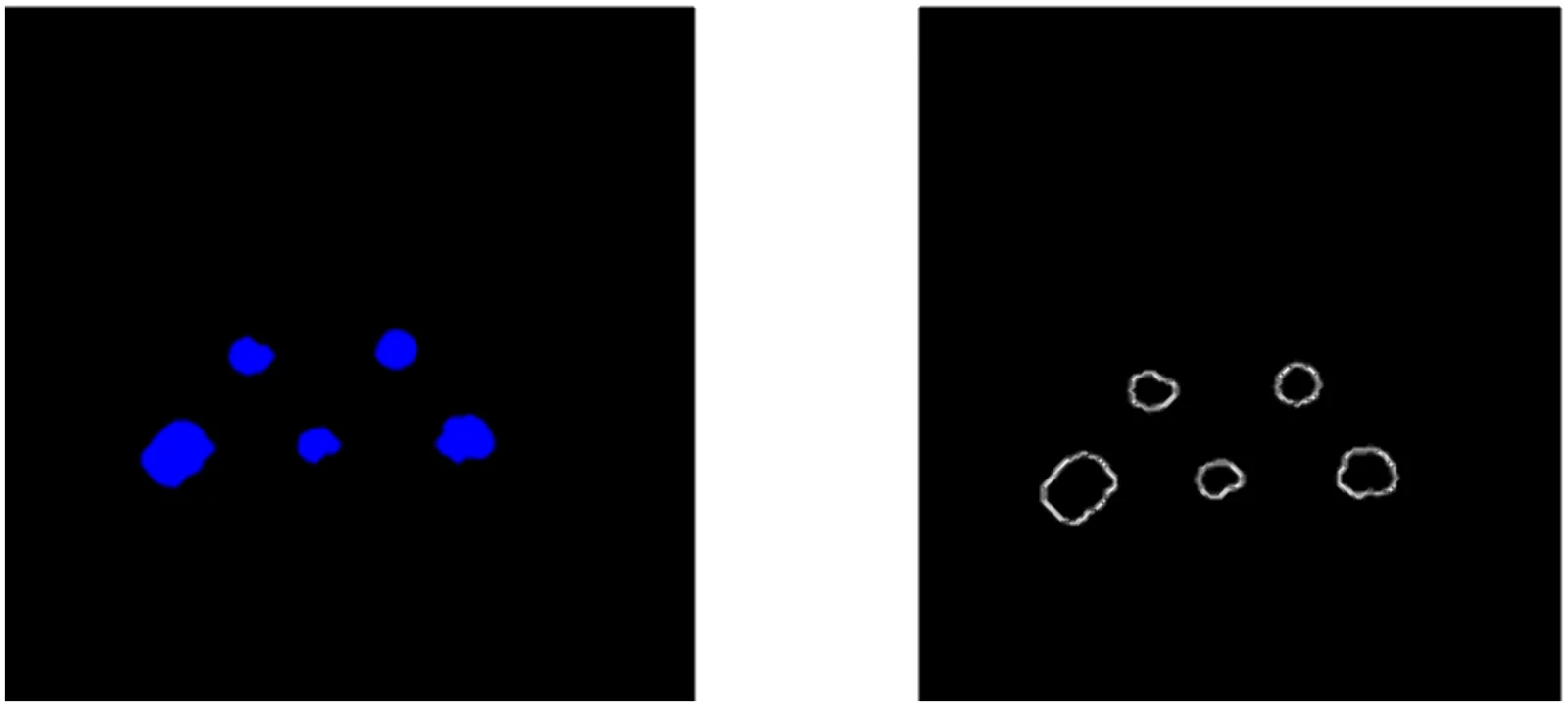
Accurate detection results: (a) image; (b) outline. (a) Accurate detection image, (b)Accurate detection image outline.

### 5.5. Smallest circle drawing

The smallest enclosing circle of the image after the previous detection step is drawn in this function. First, parameters are set, and then contour detection is performed to find the smallest enclosing circle of each contour and draw it. At the same time, the smallest enclosing circle is drawn on the initial image to verify whether the located center of the circle is accurate.

The smallest circles plotted and the overlay of the smallest circles on the source image are shown in Fig 9(a) and 9(b). The found circle does not differ significantly from the location of the source hole. The localization is accurate, with only minor differences in size. In practical situations, the radius of the charging hole is known and fixed, so the radius is not used as a basis for evaluation; only the accuracy of the located circle center is used.

**Fig 9:**
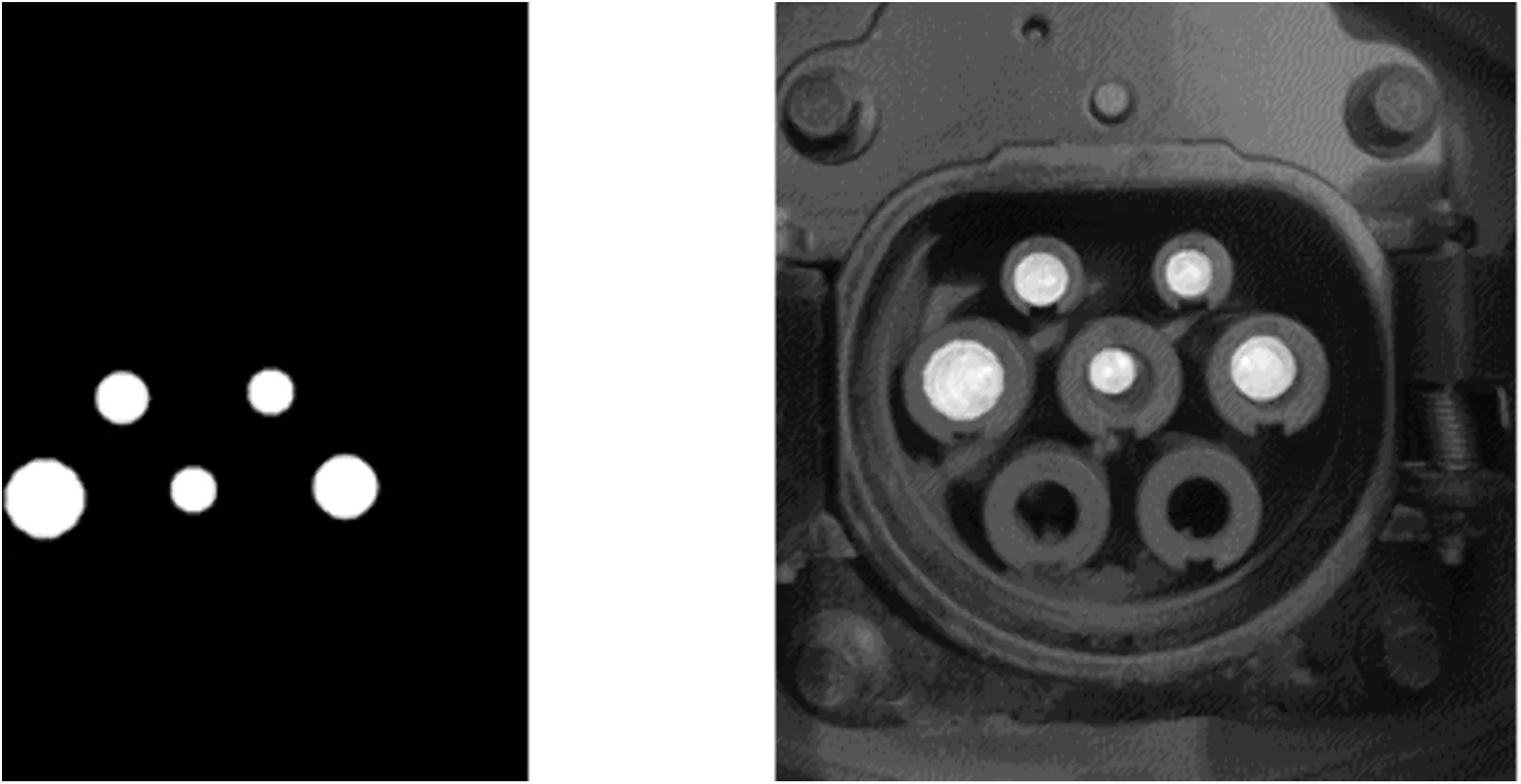
Smallest circle and overlay on source image. (a) Smallest circle image (b)Overlay of Smallest circle and source image.

### 5.6. Hough circles detection

Due to detection errors, the image drawn in the previous step does not always contain the five circular holes required; in some cases two circles appear at positions where there should have been one circle (i.e., multiple smallest circles). It is therefore necessary to perform Hough circle detection to compute the true circle centers.

First, the relevant parameters are defined; then detection is performed. After detection, the algorithm checks whether the number of detected circles is five. If not, the parameters are adjusted automatically and detection is repeated until the number of detected circles equals five. Different starting parameters may be required for images of different sizes. The default values, search ranges, and selection rationale of the Hough verification parameters are reported together with all other parameters in Table 1 (Section 5.7), and the stability of the framework with respect to these parameters is reported in Section 5.7 as well.

After detection is completed, the circles are drawn from the detected information and overlaid on the source image, as shown in Fig 10. The charging hole has been identified.

**Fig 10:**
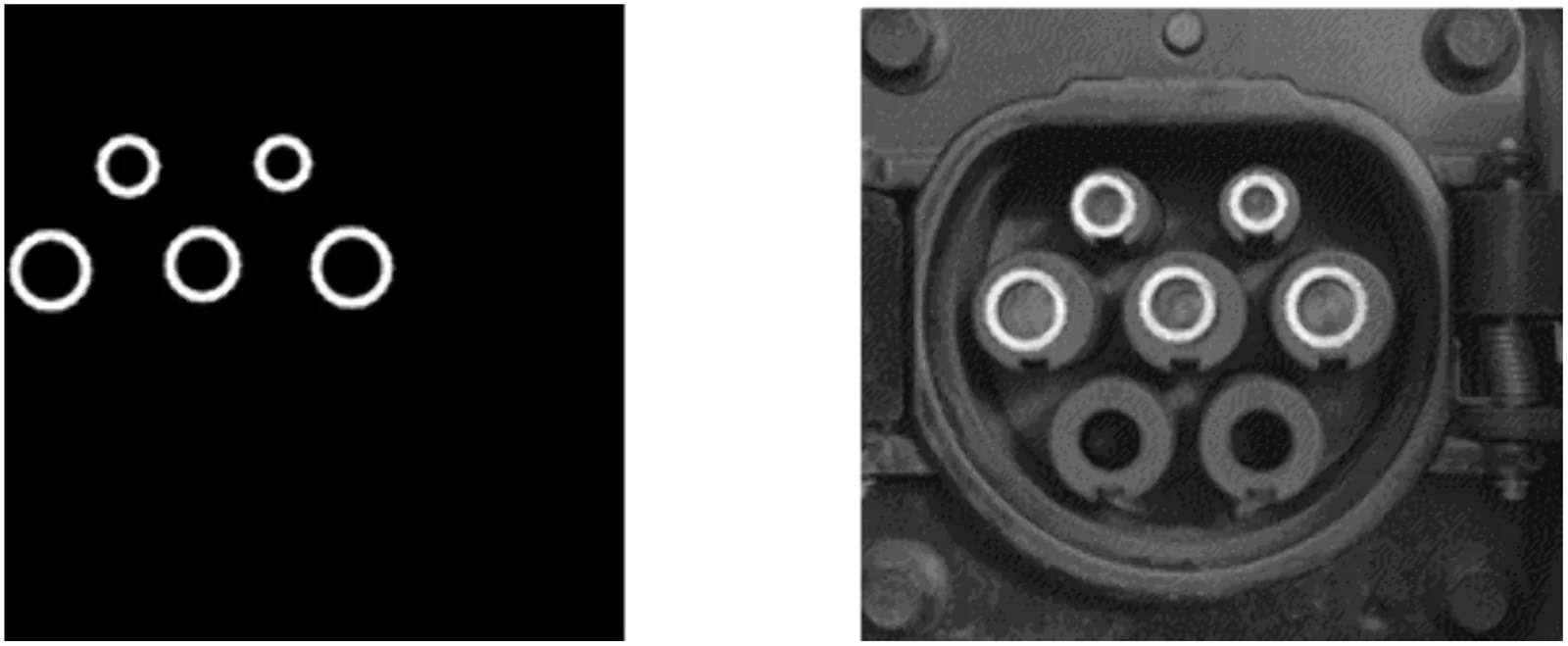
Hough circle test results. (a) Hough Circle detected circle (b) Overlay of Hough circle and source image.

### 5.7. Parameter design and perturbation analysis

Parameter design is an important part of the proposed framework. The previous subsections describe the role of each parameter at the corresponding stage of the pipeline. To improve reproducibility and to make the engineering rationale of the framework explicit, this subsection consolidates all the key parameters into a single table (Table 2) and reports a parameter perturbation analysis (Table 2) that quantifies how the localization accuracy and detection success rate respond to small changes around the default parameter setting.

**Table 2:** Parameter perturbation analysis of the proposed charging hole localization framework.

Parameter	Setting	Mean error (pixels)	Success rate (%)
Gaussian blur kernel	3 × 3	4.2	96.25
Gaussian blur kernel	5 × 5	4.8	95.00
HSL lightness factor	1.10	5.0	93.75
HSL lightness factor	1.20	4.2	96.25
HSL lightness factor	1.30	4.6	95.00
Contour thickness	1 px	5.7	91.25
Contour thickness	2 px	4.2	96.25
Contour thickness	3 px	4.9	95.00
Hough threshold	16	4.7	95.00
Hough threshold	18	4.2	96.25
Hough threshold	20	5.4	92.50

Because the proposed framework is deterministic for a fixed input image and a fixed parameter configuration (see Section 3), the conventional notion of evaluating optimization stability across stochastic re-runs does not directly apply. The relevant question is instead the stability of the framework with respect to the chosen parameter configuration. To answer this question, we performed a parameter perturbation analysis: starting from the default parameter setting, we perturbed several key parameters that directly affect different stages of the pipeline, namely the Gaussian blur kernel size (3 × 3 and 5 × 5), the HSL lightness factor (1.10, 1.20, 1.30), the contour line thickness (1, 2, 3 px), and the Hough accumulator threshold (16, 18, 20). For each perturbation, all other parameters were held at their default values, and the entire dataset was processed by the resulting pipeline. The mean localization error and the detection success rate were measured. The results are reported in Table 2.

Across all perturbations explored in Table 2, the mean localization error remained within 4.2–5.7 pixels, and the detection success rate remained within 91.25%–96.25%. This indicates that the proposed framework is not overly sensitive to minor changes in any single parameter and that the selected default settings provide a good balance between localization accuracy and detection stability. In particular, the default settings (Gaussian 3 × 3, lightness 1.20, contour thickness 2 px, Hough threshold 18) achieve the lowest mean error (4.2 px) and the highest success rate (96.25%) in this perturbation grid, which corroborates the parameter selection rationale summarized in Table 1.

## 6. Localization performance and condition-specific ablation

This section reports the localization performance of the proposed framework. Because the dataset deliberately covers four practical conditions (normal illumination, low-light, reflective/ambiguous, and mild viewpoint variation), the evaluation is reported separately for each condition, in addition to the overall result. This condition-specific reporting makes it possible to link each component of the framework to the type of image difficulty it is designed to mitigate, in line with the design intuitions described in Sections 3–5.

### 6.1. Evaluation protocol

For each image, the ground-truth centers of the five charging holes were annotated manually under the supervision of the authors. The localization error of an image is defined as the mean Euclidean distance, in pixels, between the five estimated centers and their corresponding ground-truth centers. The detection success rate is defined as the fraction of holes for which the estimated center is within a tolerance of one charging-hole radius from the ground-truth center; this tolerance corresponds to the practical success criterion of the downstream insertion stage.

### 6.2. Condition-specific ablation

To attribute the observed performance to specific design choices, we compare the full framework against four ablated variants, each of which removes one component of the pipeline: (i) w/o HSL adjust., in which the parameterized HSL adjustment of Section 5.3 is disabled and the HSL image is used directly; (ii) w/o two-stage contour, in which only the quick detection step of Section 5.4.1 is used and the accurate detection step of Section 5.4.2 is skipped; (iii) w/o Hough verif., in which the Hough verification of Section 5.6 is removed and the smallest-circle centers from Section 5.5 are taken as the final output; and (iv) w/o exception handling, in which the exception-handling rules of Section 7 (image superposition and parameter adjustment for multiple smallest circles) are disabled. For each variant, the entire dataset is processed and the per-condition localization error and detection success rate are computed. The results are reported in Table 3.

**Table 3:** Condition-specific localization performance of the proposed framework and selected ablated variants (mean error in pixels/ detection success rate in %).

Condition	Full framework	w/o HSL adjust.	w/o two-stage contour	w/o Hough verif.	w/o exception handling
Normal illumination	2.8/ 100.00	3.4/ 100.00	3.6/ 95.00	4.0/ 95.00	3.2/ 100.00
Low-light	4.5/ 95.00	8.2/ 80.00	6.8/ 85.00	6.0/ 90.00	5.6/ 85.00
Reflective/ ambiguous	5.5/ 90.00	9.0/ 75.00	8.1/ 80.00	8.8/ 80.00	7.7/ 75.00
Mild viewpoint variation	4.0/ 100.00	5.6/ 90.00	5.9/ 85.00	6.6/ 85.00	4.8/ 95.00
**Overall**	**4.2/ 96.25**	**6.6/ 86.25**	**6.1/ 86.25**	**6.4/ 87.50**	**5.3/ 88.75**

### 6.3. Discussion

Several observations follow from Table 3. First, the full framework consistently outperforms every ablated variant under every condition, and its advantage becomes more pronounced under challenging conditions (low-light, reflective/ambiguous). For example, under reflective/ambiguous conditions, the full framework reaches 5.5 px mean error and 90.00% success rate, compared with 9.0 px/ 75.00% for the variant without HSL adjustment. This supports the design intuition that HSL adjustment is particularly useful when the visual contrast between the charging holes and the surrounding metallic surface is reduced by reflections or ambiguous geometric features.

Second, the two-stage contour detection contributes most when the contours are coarse or fragmented; this is reflected in the gap between the full framework and the w/o two-stage contour variant under low-light (4.5 vs 6.8 px) and reflective/ambiguous (5.5 vs 8.1 px) conditions, where preliminary contours are noisier.

Third, the Hough verification stage is especially helpful for resolving ambiguous geometric candidates: removing it costs about 1.5–3.3 px under non-trivial conditions and lowers the success rate by 5–10 percentage points. This is consistent with its role as the last stage that enforces the geometric prior of five circular holes.

Fourth, the exception handling rules are most visible under conditions in which detections are unstable or incomplete: under low-light and reflective/ambiguous conditions, removing exception handling increases the mean error by about 1–2 px and lowers the success rate by 10–15 percentage points relative to the full framework. Under normal illumination, in contrast, the exception handling is rarely triggered and its absence has only a small effect.

Taken together, these results support the claim that the proposed framework is not only accurate under standard conditions but also robust under practically challenging conditions, and that each of its main components contributes a distinct, identifiable benefit.

### 6.4. Computational efficiency

On the implementation environment described in Section 4.2 (Intel Core i7-12700, 16 GB RAM, Windows 10 64-bit, Python with OpenCV, no GPU acceleration), the proposed framework processes each image in approximately 60 ms on average. This figure refers to the algorithmic pipeline (preprocessing, HSL conversion and adjustment, contour-based detection, geometric fitting, and Hough verification) and excludes image acquisition, external communication delay, and the mechanical motion time of the charging robot. Achieving this throughput on a conventional CPU and without any GPU acceleration confirms the lightweight and engineering-deployable nature of the proposed framework, and supports its applicability to real-time or near-real-time automatic charging operation.

## 7. Exception handling

### 7.1. Sparse image information

In practice, it has been observed that limited input image information may lead to greater error in contour detection. The solution adopted in this paper is to change the parameters of the HSL spatial adjustment function to generate different versions of the image, then to superimpose these versions and perform contour detection on the result.

### 7.2. Multiple smallest circles

In the smallest-circle detection step, multiple smallest circles may be detected, as shown in Fig 11.

**Fig 11:**
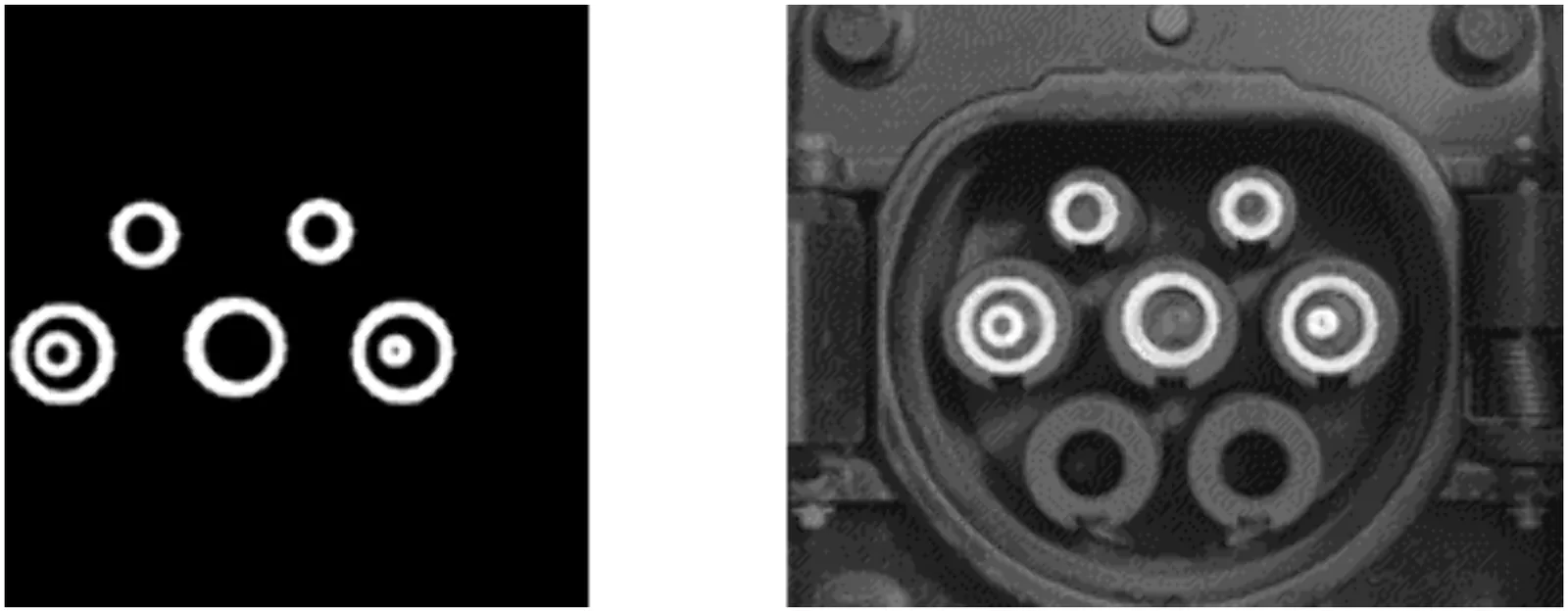
Multiple smallest circles. (a) Multiple Smallest circles (b) Overlay of multiple smallest circles and source image.

This problem is solved by changing the parameters in the experiment. The choice is changed to reduce the detected radius of the circle (for example, by setting it to the average of the actual radius), and to increase the thickness of the line (for example, by setting it to the radius of the circle). This produces a complete circular surface, with small circles surrounded without redundant gaps. The disadvantage of this approach is that the circle centers detected at this stage cannot be directly used; it is therefore necessary to perform Hough circle detection to obtain usable circle centers.

## 8. Conclusion and discussion

Based on a self-designed automatic charging system, a precise localization method for charging holes is introduced in this paper. The method is based on machine vision: it processes the captured image and finally identifies and locates the charging hole through five steps – filtering, color-space conversion, HSL color adjustment, contour detection, and Hough circle detection. Some abnormal-case handling and parameter settings are also explained.

On a self-collected dataset of 80 images acquired from six vehicles equipped with a five-hole charging interface and covering four practical conditions, the proposed framework achieves an overall mean localization error of 4.2 pixels and a 96.25% detection success rate, with an average per-image runtime of approximately 60 ms on an Intel Core i7-12700 CPU without GPU acceleration. The condition-specific ablation analysis reported in Table 3 further shows that each main component of the framework (HSL adjustment, two-stage contour detection, Hough verification, and exception handling) provides a distinct and identifiable benefit, particularly under challenging conditions such as low-light and reflective/ambiguous scenes.

Scope and generalizability. We explicitly note that the experimental validation in this paper is conducted on one charging port type with five circular holes, and that the implementation is configured accordingly. In particular, the verification stage uses an interface-specific geometric prior that the algorithm should detect exactly five circles in the expected spatial configuration. The remainder of the pipeline (image filtering, HSL-based enhancement, contour-driven region extraction, minimum enclosing circle fitting, and Hough verification) is, however, generic. The proposed method should therefore be understood as a general contour-driven localization framework combined with a target-specific geometric constraint. To extend the framework to other charging standards with different hole counts or layouts, the verification stage would need to be reconfigured by modifying the expected number of hole candidates, the allowable radius range, and the relative spatial relationships among the detected circles. The contour-driven backbone can remain unchanged. We do not claim universal plug-and-play generalization across all charging standards without modification, and we explicitly mark this as a limitation of the present implementation.

Future research will be conducted in two directions: attempting to use neural networks to generate multiple samples to reduce errors due to image simplification; and studying the localization of charging holes in complex environments (cloudy or rainy weather, night, dirt, blocked charging holes, etc.). In addition, we plan to investigate the extension of the present framework to charging interfaces with different standards and layouts, by redesigning the interface-specific geometric verification module while preserving the same contour-driven localization backbone.
