# Supplementary material for: A machine-vision framework for electric vehicle charging hole localization based on contour-driven detection and geometric verification
Source: PLoS One. 2026 Oct 22;21(10):e0359570. doi: 10.1371/journal.pone.0359570 (PMC13690247; doi:10.1371/journal.pone.0359570)
Supplement: Supplementary file 1 [file pone.0359570.s002.docx]

**Response Letter**

**Paper ID:** PONE-D-26-05627
**Title:** *A Machine-Vision Framework for Electric Vehicle Charging Hole Localization Based on Contour-Driven Detection and Geometric Verification*
**Authors:** Wei Luo, Qiantao Huo, and Xin Li

The authors would like to thank the Academic Editor and the anonymous reviewers for their valuable and constructive comments. We have carefully revised the manuscript according to all the comments. These comments are greatly helpful for improving the quality, clarity, and reproducibility of our work. In this response letter, we provide a point-by-point response to each comment. All modifications have been incorporated into the revised manuscript and highlighted in the marked-up version.

**Summary of Major Revisions**

Before responding to each comment in detail, we provide here a high-level summary of the major changes made in the revised manuscript. The list below maps the principal revisions to the comment(s) that motivated them, so that the editor and the reviewers can quickly assess the overall scope of the revision. Detailed point-by-point responses are provided in the subsequent sections.

1. **Dataset and image-acquisition setup (R1-1, R2-1, R2-2).** The description of the self-collected dataset has been substantially expanded, including the number of vehicles (six), the charging interface type (one five-hole interface), the camera configuration (monocular RGB camera mounted on the charging robot end-effector, 1920×1080), and the realistic variations represented in the data (illumination, viewing angle, reflective interference, background complexity). A new figure with representative samples under different practical conditions has been added.
2. **System workflow and the role of perception (R2-3).** The system workflow subsection has been clarified to make explicit that the proposed framework operates at the precise positioning stage on images acquired at a designated observation pose after coarse positioning, rather than as a continuous visual servoing module during arbitrary motion.
3. **Comprehensive parameter table and parameter perturbation analysis (R1-2, R1-6).** A consolidated parameter table (Table 2) has been added, listing all key parameters with default values, tuning ranges, and selection rationale for each stage of the pipeline. A parameter perturbation analysis (Table 5) has also been added, evaluating the stability of the framework around the default parameter setting.
4. **Condition-specific ablation analysis (R2-4).** The ablation analysis has been reorganized to report results separately under different image conditions (normal illumination, low-light, reflective/ambiguous, mild viewpoint variation), so that the contribution of each component can be linked to the corresponding type of image difficulty. The new condition-specific results are reported in Table 6.
5. **Hardware and runtime details (R1-5).** The computational efficiency subsection has been expanded with the CPU model, memory size, operating system, software environment, and the explicit statement that no GPU acceleration was used. The scope of the reported 60 ms runtime has also been clarified.
6. **Scope and generalizability of the framework (R1-3).** The discussion has been revised to explicitly state that the current implementation is configured for a five-hole charging interface, while the overall pipeline (preprocessing, HSL adjustment, contour detection, minimum enclosing circle, Hough verification) is generic. A discussion of how the framework can be extended to other charging standards by reconfiguring the geometric verification module has been added, together with a corresponding limitation statement.
7. **Related-work expansion (R1-4, ER6).** The Introduction has been enriched with the suggested references on EV charging-system resilience and monitoring, while the technical scope of the present work (vision-based charging-hole localization) is preserved.
8. **Data availability statement (ER2, R2-5).** The Data Availability Statement has been updated to transparently describe that the underlying image data are subject to confidentiality restrictions arising from a commercial collaboration agreement. The derived numerical materials needed to interpret the reported results (summary statistics, per-condition results, parameter perturbation results, and the complete parameter configuration) are included directly in the revised manuscript.
9. **Funding statement and acknowledgments (ER3).** Funding-related text has been removed from the Acknowledgments section, and the Funding Statement to be entered in the submission system is provided in the response to ER3.
10. **Manuscript formatting and Figure 3 (ER1, ER4, ER5).** The manuscript has been reformatted according to the PLOS ONE Word manuscript style requirements; Figure 3 has been replaced by a newly created original figure produced entirely by the authors to fully comply with the CC BY 4.0 license.

All changes have been marked in the revised manuscript. The detailed point-by-point responses are provided in the following sections.

**Responses to Academic Editor and Journal Requirements**

We sincerely thank the Academic Editor for the careful handling of our manuscript and for the opportunity to revise and improve it. The detailed responses to each Journal Requirement are provided below.

**Editorial Requirement 1:** Please ensure that your manuscript meets PLOS ONE's style requirements, including those for file naming.

**Response:** Thank you for this reminder. We have carefully checked and revised the manuscript to comply with the PLOS ONE style requirements, including manuscript structure, formatting, and file naming. The revised files have been prepared accordingly.

**Editorial Requirement 2:** Please confirm whether the submission contains all raw data required to replicate the results of the study. If not, please upload them as Supporting Information files or deposit them in a public repository.

**Response:** We appreciate the editor’s important comment regarding data availability. We would like to clarify the situation transparently. The image data used in this study were acquired on a real electric vehicle charging platform that was developed under a commercial collaboration with industrial partners. As a consequence, the underlying raw image data and the associated annotations are subject to confidentiality obligations agreed upon with the partner company, and they cannot be deposited in a public repository or released as Supporting Information. This restriction is consistent with the response we provide to Reviewer 2 (Comment 5).

That said, in order to support reproducibility of the reported findings to the maximum extent possible within these restrictions, the revised manuscript itself directly contains the derived numerical materials needed to interpret and replicate the results, including:

- the numerical values behind the summary statistics, means, and success rates reported in the tables and figures;
- the per-condition localization performance used to construct the condition-specific analysis;
- the parameter perturbation results used to evaluate robustness around the default parameter setting; and
- the complete parameter configuration of the proposed framework, including default values, candidate ranges, and design rationale for each stage of the pipeline.

We have correspondingly revised the Data Availability Statement of the manuscript to explicitly describe (a) that the underlying image data are restricted due to a commercial collaboration agreement with industrial partners; (b) that the partner company has imposed these restrictions; and (c) the contact information through which qualified researchers may submit access requests, with any potential access remaining subject to approval by the relevant collaborating parties and the terms of the underlying agreement. We hope this resolution is acceptable to the editorial office.

**Editorial Requirement 3:** Funding information appears in the Acknowledgments section but is inconsistent with the Funding Statement in the submission form.

**Response:** Thank you for pointing this out. We have revised the manuscript accordingly. Specifically, we have removed the funding-related text from the Acknowledgments section. We would like to update the Funding Statement in the submission system to the following:

**Funding Statement:**

*This work was supported by the Jiangsu Province College Students' Innovation and Entrepreneurship Training Program Project of China (Grant No. 201913654021Y).*

Please kindly update the online submission form on our behalf.

**Editorial Requirement 4:** Please update your submission to use the PLOS LaTeX template.

**Response:** Thank you for the instruction. We would like to respectfully clarify that the present manuscript was originally prepared and submitted in Word (.docx) format rather than LaTeX, and PLOS ONE accepts manuscripts in either Word or LaTeX format (see the PLOS ONE Submission Guidelines, “File Formats and Templates” section, available at https://journals.plos.org/plosone/s/submission-guidelines). Accordingly, we have reformatted the revised manuscript strictly according to the PLOS ONE Word manuscript style requirements, including the structure of the title page, author affiliations, headings, figure and table captions, in-text citation style, and the Reference list format. The revised .docx files have been prepared and uploaded accordingly. We hope this resolution is acceptable to the editorial office; if a LaTeX version is preferred, we are of course happy to convert the manuscript to the PLOS LaTeX template upon request.

**Editorial Requirement 5:** Figure 3 appears to contain copyrighted images.

**Response:** Thank you for this important comment, and we apologize for the oversight. We have carefully reviewed Figure 3 and confirm that, in order to fully comply with the CC BY 4.0 license requirement of PLOS ONE, we have replaced the previous Figure 3 with a newly created original figure produced entirely by the authors. The new figure is generated from images and diagrams owned by the authors and is therefore unambiguously free of any third-party copyright restrictions. The figure caption has been updated accordingly to reflect that the figure is original work created by the authors. No third-party copyright permission form is therefore required.

**Editorial Requirement 6:** If the reviewer comments include a recommendation to cite specific previously published works, please review and evaluate these publications to determine whether they are relevant and should be cited.

**Response:** Thank you. We have carefully reviewed the suggested publications. We have cited the references that are relevant to the broader context of electric vehicle charging systems and monitoring, while keeping the focus of our manuscript on machine-vision-based charging hole localization. The related discussion and citations have been added to the Introduction section of the revised manuscript.

**Responses to Reviewer 1**

**1) Comment:** The evaluation is conducted on a self-collected dataset of 80 images captured under practical conditions. Could the authors clarify how many distinct vehicles, charging port types, and environmental configurations are represented? Are all images captured from a fixed camera pose and similar geometry?

**Response:** We thank the reviewer for this important comment. We agree that the original manuscript did not provide sufficiently detailed information regarding the composition of the self-collected dataset and the image acquisition setting. Since the practical significance of the proposed framework depends not only on localization accuracy but also on the representativeness and transparency of the experimental data, we have revised the manuscript to provide a clearer and more complete description of the dataset.

Specifically, the self-collected dataset used in this study contains 80 real images acquired from 6 vehicles under practical charging conditions. All images correspond to one charging port type with five circular holes, which is the target charging interface configuration of the automatic charging system studied in this work. We would like to emphasize this point explicitly because the present study is intended to validate the proposed framework on a specific practical charging interface rather than across multiple heterogeneous charging standards. Therefore, the current dataset is interface-specific, but it still contains meaningful variability at the image level.

In terms of acquisition conditions, the images were collected on a real electric vehicle charging platform using a monocular RGB camera mounted on the charging robot end-effector, with an image resolution of 1920 × 1080 pixels. The camera intrinsic parameters were calibrated in advance. During acquisition, the camera–charging-port geometry was controlled to match the practical operating configuration of the automatic charging system, so the images were not taken from completely arbitrary poses. However, the acquisition conditions were not identical across all images. Instead, the dataset includes moderate but realistic variation in illumination level, viewing angle, reflective interference, and background complexity, which are typical factors affecting practical charging hole localization. In particular, the collected images include examples captured under normal illumination, low-light conditions, reflective surface conditions, and slightly varying viewpoints around the nominal operating pose.

To avoid any misunderstanding, we have revised the corresponding dataset description in the manuscript to make clear that the imaging geometry is controlled but not perfectly identical across all samples. This distinction is important: the dataset does not represent unconstrained in-the-wild image capture, but neither is it limited to a single repeated image configuration. Rather, it reflects the realistic operating conditions of the target charging system while maintaining a sufficiently stable geometric relationship for precise localization. These revisions have been incorporated into the dataset acquisition and experimental setup sections of the revised manuscript.

**2) Comment:** Have the authors evaluated cross-validation splits or repeated runs with parameter perturbations?

**Response:** We appreciate the reviewer’s valuable suggestion. We agree that robustness and reproducibility should be more explicitly discussed. At the same time, we would like to clarify that the proposed method is fundamentally different from a learning-based model, and therefore the interpretation of cross-validation and repeated runs should be adjusted accordingly.

Specifically, the proposed method is a rule-based and geometry-driven machine-vision framework, rather than a trainable model involving stochastic optimization. It does not contain random weight initialization, iterative training, or data-driven parameter fitting. Instead, the localization result is obtained through a deterministic sequence of image filtering, RGB-to-HSL conversion, HSL-based enhancement, contour-driven detection, minimum enclosing circle fitting, and Hough circle verification. Therefore, for a fixed input image and a fixed parameter configuration, the algorithm always produces the same output. For this reason, repeated runs in the conventional sense used for deep learning methods are not directly applicable here, and k-fold cross-validation is also not the primary validation protocol for a deterministic vision pipeline of this type.

However, we fully agree with the reviewer that the stability of the method with respect to parameter choice is an important issue, especially because parameter design is emphasized as part of the methodological contribution. In response to this comment, we have strengthened the manuscript in two respects. First, we have clarified in the revised manuscript that the proposed framework is deterministic and does not involve stochastic training variability. This helps distinguish the present work from trainable models for which repeated runs are necessary to assess optimization instability. Second, and more importantly, we have added a small parameter perturbation analysis to evaluate the robustness of the framework around the default parameter setting. Specifically, we perturbed several key parameters that directly affect different stages of the pipeline, including: 1) the Gaussian blur kernel size (3×3 and 5×5), 2) the HSL lightness adjustment factor (1.10, 1.20, and 1.30), 3) the contour thickness (1, 2, and 3 pixels), and 4) the Hough accumulator threshold (16, 18, and 20), while keeping the other parameters fixed.

The results show that the proposed framework remains stable within a reasonable neighborhood of the selected default setting. Across these perturbations, the mean localization error and the detection success rate remain within tight bounds, as reported in the table below. These results indicate that the proposed method is not overly sensitive to minor parameter changes and that the selected default settings provide a good balance between localization accuracy and detection stability. We believe this analysis addresses the reviewer’s concern in a way that is more appropriate for the nature of the present method than conventional repeated stochastic runs. We have added the corresponding discussion and results to the revised manuscript and also summarized the key parameter settings in a dedicated parameter table to further improve reproducibility.

**Table 1. Parameter perturbation analysis of the proposed charging hole localization framework.**

| **Parameter** | **Setting** | **Mean error (pixels)** | **Success rate (%)** |
| --- | --- | --- | --- |
| Gaussian blur kernel | 3×3 | 4.2 | 96.25 |
| Gaussian blur kernel | 5×5 | 4.8 | 95.00 |
| HSL lightness factor | 1.10 | 5.0 | 93.75 |
| HSL lightness factor | 1.20 | 4.2 | 96.25 |
| HSL lightness factor | 1.30 | 4.6 | 95.00 |
| Contour thickness | 1 px | 5.7 | 91.25 |
| Contour thickness | 2 px | 4.2 | 96.25 |
| Contour thickness | 3 px | 4.9 | 95.00 |
| Hough threshold | 16 | 4.7 | 95.00 |
| Hough threshold | 18 | 4.2 | 96.25 |
| Hough threshold | 20 | 5.4 | 92.50 |

**3) Comment:** Different electric vehicles use different charging standards and geometries. The method assumes detection of exactly five circular holes. How does the framework generalize to charging interfaces with different hole counts or layouts? Is the detection logic hard-coded for five holes?

**Response:** We thank the reviewer for raising this important point. We fully agree that the scope and generalizability of the proposed framework should be more explicitly discussed, especially because charging interfaces of different electric vehicle standards may vary in terms of hole count, geometric layout, and structural configuration.

In the current study, the implemented system is indeed designed for one specific charging interface type containing five circular holes, and the present detection logic is configured accordingly. In particular, the current implementation includes an interface-specific verification rule in both the quick detection stage and the final Hough-based verification stage, where the algorithm checks whether exactly five candidate regions/circles are obtained. Therefore, to answer the reviewer’s question directly, the current implementation is application-specific at the geometric verification level, and the expected number of holes is indeed incorporated into the present decision logic.

We have revised the manuscript to state this point more explicitly, because in the original version this assumption was embedded in the algorithm description but was not sufficiently discussed as a scope condition. We agree that this clarification is important to prevent the reader from overinterpreting the current implementation as a fully universal detector for arbitrary charging interfaces. At the same time, we would like to emphasize that the overall methodological framework is not fundamentally restricted to five-hole interfaces. The core pipeline of the proposed method consists of image preprocessing, HSL-based enhancement, contour-driven region extraction, minimum enclosing circle fitting, and geometric verification. These stages are generic in the sense that they are designed to extract and refine circular charging-hole candidates from the image and estimate their geometric centers. What is interface-specific in the present study is not the entire framework, but rather the geometric prior used in the final verification stage.

In other words, the current method should be understood as a general contour-driven localization framework combined with a target-specific geometric constraint. For the charging interface studied in this work, the appropriate geometric prior is that five circular holes should be detected in the expected spatial configuration. For another charging standard, the same overall pipeline could still be applied, but the final verification logic would need to be reconfigured accordingly. This reconfiguration may include modifying: the expected number of hole candidates, the allowable radius range, the relative spatial relationships among the detected circles, and the corresponding consistency criteria used to reject ambiguous detections. Therefore, the current work does not claim universal plug-and-play generalization across all charging standards without modification. Rather, its contribution lies in presenting a lightweight, interpretable, and engineering-oriented machine-vision framework that can reliably localize charging holes when appropriate structural priors of the target interface are incorporated. We believe this is a more accurate and scientifically rigorous description of the current work.

To make this clearer, we have added a corresponding discussion in the revised manuscript and explicitly acknowledged that the current experimental validation is conducted on one charging port type with five circular holes. We have also included this point as a limitation of the present study and as an important direction for future work. Specifically, future work will investigate extending the current framework to charging interfaces with different standards and layouts by redesigning the interface-specific geometric verification module while preserving the same contour-driven localization backbone. We believe this revision more clearly distinguishes between the generic part of the framework and the application-specific geometric prior, and therefore better addresses the reviewer’s concern about generalizability.

**4) Comment**: To enrich the manuscript, more related works could be reviewed such as Generative self-supervised learning for cyberattack-resilient EV charging demand forecasting, 10.1109/TITS.2025.3545577; Toward resilient electric vehicle charging monitoring systems: Curriculum guided multi-feature fusion transformer, 10.1109/TITS.2024.3456843

**Response:**
We appreciate the reviewer’s thoughtful recommendation. We agree that positioning the present study more clearly within the broader context of electric vehicle charging systems can help improve the manuscript.

We carefully reviewed the suggested work. These studies are relevant to the broader domain of electric vehicle charging infrastructure, especially in terms of charging-system resilience, monitoring, and intelligent operation under complex conditions. Although their primary focus is different from that of our work, they are still valuable in providing a wider technological background for automatic charging-related systems. In contrast, the present manuscript focuses specifically on a machine-vision-based charging hole localization problem, whose goal is to accurately identify and localize the geometric centers of charging holes to support the physical alignment and insertion stage of an automatic charging system. Therefore, the technical scope of our work is more centered on vision-based perception and geometric localization, rather than charging demand forecasting, cyberattack resilience, or system-level charging monitoring.

To address the reviewer’s suggestion in a balanced manner, we have revised the Introduction section to incorporate the recommended references where appropriate. Specifically, we cite these studies as representative examples of the broader research efforts aimed at improving the intelligence, resilience, and operational reliability of electric vehicle charging systems. At the same time, we preserve the distinction between those works and the present study by clarifying that our contribution lies in the precise localization of charging holes using a lightweight and interpretable machine-vision framework, rather than in forecasting, monitoring, or transformer-based charging system analytics. We believe this revision improves the contextual completeness of the manuscript without diluting its main technical focus. The corresponding references and related discussion have been added to the revised Introduction section.

**5) Comment:** The runtime is reported as 60 ms per image. However, hardware details are limited. Please specify CPU model, memory, and whether GPU acceleration was used.

**Response:** We thank the reviewer for this helpful comment. We agree that the runtime reported in the original manuscript would be more meaningful and reproducible if the underlying hardware and software environment were described more explicitly.

In the original manuscript, we reported that the proposed framework processes each image in approximately 60 ms, indicating that the method is suitable for real-time or near-real-time automatic charging applications. However, we acknowledge that without a clear description of the computing platform, this number is difficult for readers to interpret or compare with other methods. Since the proposed framework is intended as a lightweight and practically deployable engineering solution, it is particularly important to specify whether the reported runtime depends on specialized hardware acceleration or can be achieved using ordinary computing resources.

Accordingly, in the revised manuscript, we have added the detailed hardware and execution environment used for runtime evaluation, including the CPU model, memory size, operating system, implementation environment, and whether GPU acceleration was used. Specifically, the experiments were conducted on an Intel Core i7-12700 CPU, with 16 GB RAM, under Windows 10 64-bit, and implemented in Python with OpenCV. No GPU acceleration was used in the reported runtime experiments. This clarification is important because it shows that the reported efficiency of the proposed framework is achieved using a conventional CPU-based environment rather than relying on a dedicated high-end GPU. As a result, the runtime result better reflects the lightweight and deployment-friendly nature of the proposed method.

In addition, we have clarified what is included in the reported runtime. Specifically, the reported average processing time of approximately 60 ms per image refers to the image processing and localization pipeline itself, including preprocessing, HSL conversion and adjustment, contour-based detection, geometric fitting, and Hough verification. It does not include image acquisition from the camera, external communication delay, or the mechanical motion time of the charging robot. We believe this clarification helps distinguish the algorithmic runtime from the overall execution time of the full charging system, which may additionally depend on hardware communication and motion control.

We have added these details to the computational efficiency subsection of the revised manuscript. This revision improves the interpretability and reproducibility of the reported runtime and further supports our claim that the proposed framework achieves a good balance between localization accuracy, robustness, and practical efficiency.

**6) Comment:** Since parameter design is emphasized as a contribution, the manuscript should provide a comprehensive table listing all key parameters, default values, tuning ranges, and selection rationale.

**Response:** We appreciate this very constructive suggestion and fully agree with the reviewer. Since parameter design and engineering configuration are emphasized as an important part of the proposed framework, the manuscript should provide a clearer and more systematic summary of the key parameters used in different stages of the method.

In the original manuscript, many important parameter settings were already described throughout the method section, but they were scattered across multiple subsections. For example, the image preprocessing stage discussed different filtering methods and kernel sizes; the HSL adjustment stage described the use of parameterized enhancement to improve contrast; the contour detection stage provided specific values for contour line thickness, circle thickness, and the initial minimum circle radius; and the Hough verification stage explained that the parameters were iteratively adjusted until the desired geometric configuration was obtained. While these descriptions conveyed the design logic of individual components, we agree that presenting them only in distributed form makes it difficult for readers to quickly grasp the full parameter configuration of the framework or to reproduce the implementation efficiently.

To address this issue, in the revised manuscript we have added a new table that systematically summarizes the key parameters of the proposed framework, including their default values, candidate/tuning ranges, functional roles, and selection rationale. The table covers the main parameters involved in: image filtering, HSL-based enhancement, contour extraction and drawing, minimum enclosing circle fitting, and Hough circle verification. In particular, the added table helps clarify that the selected parameters were not chosen arbitrarily, but were designed according to the practical characteristics of the charging-hole localization task. For example, some parameters are intended to suppress noise while preserving circular structure, some are designed to ensure adequate filling of candidate hole regions, and others are used to balance detection completeness and geometric discrimination during verification. By consolidating these settings into a single table, the revised manuscript more clearly reflects the engineering rationale of the proposed framework and makes the contribution of parameter design more explicit.

In addition, this new table complements the newly added parameter perturbation analysis. Together, the two additions provide both: a static view of the selected parameter configuration and its rationale, and a dynamic view of how the framework behaves under reasonable variations of several key parameters. We believe this significantly improves the clarity, reproducibility, and engineering transparency of the manuscript. The complete parameter table has been added to the method/experimental analysis section of the revised manuscript as Table 2, “Key parameter settings of the proposed charging hole localization framework,” and the parameter perturbation analysis is reported in Table 5. For the convenience of the reviewer, the same parameter table is reproduced below, with the values used in the implementation.

**Table 2. Key parameter settings of the proposed charging hole localization framework.**

| **Stage** | **Parameter** | **Default** | **Tuning range** | **Selection rationale** |
| --- | --- | --- | --- | --- |
| Filtering | Filter type | Gaussian | Mean / Gaussian / Median | Suppress noise while preserving circular contour structure. |
| Filtering | Convolution kernel | 3×3 | 3×3 / 5×5 | Balance smoothing strength against blurring of small charging-hole features. |
| HSL adjustment | Lightness factor | 1.20 | 1.10–1.30 | Improve separability of dark charging holes from background under varying illumination. |
| HSL adjustment | Saturation factor / Hue offset | 1.10 / 0° | 0.90–1.20 / −5°–5° | Enhance contrast between charging-hole region and surrounding metallic surface while avoiding excessive color distortion. |
| Quick contour detection | Contour line thickness | 2 px | 1–3 px | Ensure connected contours of candidate hole regions. |
| Quick contour detection | Drawn-circle line thickness | 12 px | 8–16 px | Fill the candidate hole region without spilling into adjacent regions. |
| Min. enclosing circle | Initial smallest-circle radius | R/2 | 0.4R–0.6R | Provide a stable seed radius relative to the known charging-hole physical size. |
| Min. enclosing circle | Min.-circle line thickness | R | 0.8R–1.2R | Eliminate residual gaps inside the candidate region for stable subsequent verification. |
| Hough verification | Accumulator threshold | 18 | 16–20 | Balance true-circle detection rate against false circles in cluttered regions. |
| Hough verification | Min / Max circle radius | 22 / 45 px | 18–50 px | Constrain detection to the physically plausible charging-hole radius range in the captured images. |
| Hough verification | Expected number of holes | 5 | Interface-specific | Geometric prior of the target five-hole charging interface. |

**Responses to Reviewer 2**

**1) Comment:** It will be more helpful if more information on data is included, for example, if variations of conditions (different illumination, or location) can be shown in a figure.

**Response:** We thank the reviewer for this helpful suggestion. We agree that, in addition to a textual description of the dataset, it is beneficial to visually show the main types of variation represented in the collected images. Since the practical value of the proposed framework depends on its performance under realistic operating conditions, providing representative image examples can make the experimental setting more transparent and easier for readers to understand.

In the revised manuscript, we have enriched the dataset description and added a new figure showing representative samples collected under different practical conditions. Specifically, the added figure includes examples corresponding to variations in illumination level, viewing location/angle, reflective interference, and background complexity, which are the main sources of visual variability encountered in our data collection. These conditions were already mentioned in the manuscript at a general level, but we agree that presenting them visually is more informative than relying on verbal description alone.

We would like to emphasize that the present dataset was collected on a real electric vehicle charging platform using a camera integrated with the charging system, rather than from idealized or artificially simplified images. Therefore, even though the current study focuses on one target charging interface type, the image set still contains meaningful practical variation at the sensing level. By adding representative examples under different conditions, the revised manuscript more clearly shows the operating scenarios under which the proposed framework was evaluated.

In addition to the new figure, we have also revised the corresponding text in the dataset description section to explicitly explain the main types of variation represented in the data and how they relate to the intended deployment scenario of the automatic charging system. We believe this revision improves the transparency of the experimental setup and provides a better visual basis for the later robustness-related discussion.

**2) Comment:** It is not clear if the camera position to capture the car charging hole is stationary, or located at the end effector.

**Response:** We appreciate the reviewer’s comment. We apologize that this part was not sufficiently clear in the original manuscript. Since the physical placement of the camera is directly related to the imaging geometry and the interpretation of the localization task, we agree that this information should be stated explicitly.

In the revised manuscript, we have clarified the camera configuration in the system description. Specifically, the images used in this study were acquired using a monocular RGB camera mounted on the charging robot end-effector. The camera resolution was 1920 × 1080 pixels, and the intrinsic parameters were calibrated in advance. This configuration was chosen because it is consistent with the intended practical operation of the automatic charging system, in which the perception module is integrated with the charging robot and directly observes the charging interface from the manipulator side.

This clarification is important for two reasons. First, it indicates that the proposed localization framework is evaluated in the same sensing configuration in which it is expected to be used in practice, rather than under a separated external camera setup. Second, it explains why the imaging geometry is relatively controlled: because the camera is physically coupled to the end-effector and operates within the charging procedure, the viewpoint is constrained by the robot’s working configuration even though some practical variation in angle and position still exists. To avoid ambiguity, we have revised the corresponding text in the system architecture and dataset acquisition sections of the manuscript to clearly state that the camera is mounted on the charging robot end-effector. We believe this clarification makes the overall system setup easier to understand.

**3) Comment:** Does the searching run while end effector is moving? or a fixed camera capture whole image, and tells end effector where it should move to?

**Response:** Thank you for this important question. We agree that the interaction between perception and execution should be more clearly described, because the role of the proposed localization framework within the overall automatic charging system may otherwise remain ambiguous.

In the revised manuscript, we have clarified the workflow of the proposed system in more detail. The proposed charging hole localization method corresponds to the precise positioning stage of the overall automatic charging framework. More specifically, the system first performs a coarse positioning step to bring the charging robot/end-effector into an appropriate observation region relative to the charging interface. After this coarse alignment, the end-effector-mounted camera captures an image of the charging port region. The proposed machine-vision-based localization framework is then applied to this captured image to estimate the geometric centers of the charging holes. These estimated positions are subsequently used by the execution/control module to guide the charging gun toward final alignment and insertion.

In other words, the precise localization stage studied in this manuscript is performed on images acquired at a designated observation pose after coarse positioning, rather than as a continuously running visual search module during arbitrary end-effector motion. We believe this clarification is important because it distinguishes the proposed method from full visual servoing during continuous motion. The present work focuses on accurate and robust localization of the charging hole geometry once the charging interface has been brought into an appropriate field of view, and this precise localization result then serves as the perception input for subsequent motion execution.

This interpretation is also consistent with the overall system description in the manuscript, where the localization process is divided into a coarse regional positioning stage and a precise positioning stage. The proposed method specifically addresses the latter. We have revised the system workflow subsection accordingly to make the sequence of coarse positioning, image acquisition, precise localization, and motion execution more explicit and easier for readers to follow. We believe this revision resolves the ambiguity regarding whether the perception runs during motion or after the robot reaches a suitable observation configuration.

**4) Comment:** In regard to ablation study, it's best to separate the analysis based on the image conditions, to prove the developed method is useful to mitigate insufficient image information or partial occlusion or ambiguous geometric features or unstable detection results.

**Response:** We greatly appreciate this valuable suggestion. We agree that condition-specific analysis can more directly support the claim that the proposed framework improves robustness under practically challenging scenarios. In the original manuscript, we reported overall ablation results and separately discussed robustness under different operating conditions. However, these analyses were not sufficiently connected, and therefore the manuscript did not fully demonstrate how the proposed components contribute under different types of image difficulty.

To address this point, in the revised manuscript we have supplemented the analysis by separating the results according to different image conditions. Specifically, we grouped the data into representative practical categories, including normal illumination, low-light conditions, reflective or visually ambiguous cases, and mild viewpoint variation. Based on these grouped conditions, we further examined the performance of the full framework and compared it with selected ablated variants. This form of analysis is more informative than reporting only a single overall average, because it allows us to observe which components are particularly important when image information is degraded or geometric ambiguity is more likely to occur.

The additional analysis shows that the complete framework consistently provides more stable and accurate localization than the simplified variants, and that this advantage becomes more evident under challenging conditions. In particular, the results support the intended design logic of the proposed framework: the HSL-based adjustment improves the separability of charging holes from the background under illumination changes; the two-stage contour detection strategy helps produce more reliable candidate regions when coarse or fragmented contours are present; the exception handling mechanisms reduce the impact of unstable or incomplete detections; and the Hough circle verification step is especially helpful for resolving ambiguous geometric candidates and improving final center estimation.

Therefore, the new condition-specific analysis more directly supports our claim that the proposed framework is not only accurate under standard conditions, but also better able to cope with insufficient image information and ambiguous geometric features in practical operation. In the revised manuscript, the corresponding results are summarized in Table 6, which reports the localization performance under different imaging conditions, and the related discussion has been expanded in the robustness analysis section. We believe this revision makes the link between the proposed design choices and the claimed robustness much clearer. For the convenience of the reviewer, the same Table 6 is reproduced below, with the values obtained in our experiments.

**Table 3. Condition-specific localization performance of the proposed framework and selected ablated variants (mean error in pixels / detection success rate in %).**

| **Condition** | **Full framework** | **w/o HSL adjust.** | **w/o two-stage contour** | **w/o Hough verif.** | **w/o exception handling** |
| --- | --- | --- | --- | --- | --- |
| Normal illumination | 2.8 / 100.00 | 3.4 / 100.00 | 3.6 / 95.00 | 4.0 / 95.00 | 3.2 / 100.00 |
| Low-light | 4.5 / 95.00 | 8.2 / 80.00 | 6.8 / 85.00 | 6.0 / 90.00 | 5.6 / 85.00 |
| Reflective / ambiguous | 5.5 / 90.00 | 9.0 / 75.00 | 8.1 / 80.00 | 8.8 / 80.00 | 7.7 / 75.00 |
| Mild viewpoint variation | 4.0 / 100.00 | 5.6 / 90.00 | 5.9 / 85.00 | 6.6 / 85.00 | 4.8 / 95.00 |
| Overall | 4.2 / 96.25 | 6.6 / 86.25 | 6.1 / 86.25 | 6.4 / 87.50 | 5.3 / 88.75 |

**5) Comment:** This journal asked all author to publish the data in open repository, so maybe author can do so and include data access information in the paper.

**Response:** We thank the reviewer for this important reminder. We fully agree that data availability is important for transparency, reproducibility, and compliance with the journal’s data policy.

However, in the present study, the data was collected as part of the development and testing of a real electric vehicle charging platform conducted under commercial collaboration with industrial partners. As a result, the underlying data, including the image data and associated evaluation data, are subject to confidentiality restrictions imposed by the collaboration agreement and the commercial interests of the partner company/companies. For this reason, the data cannot be deposited in a public repository or released as Supporting Information.

To address this issue transparently, we have revised the Data Availability Statement in the manuscript to explicitly describe the restriction, its basis, and the responsible parties. In particular, we now clarify that the data are restricted because they arise from a commercially collaborative project and are subject to confidentiality obligations agreed upon with the industrial partner(s). The Data Availability Statement also indicates that data access requests may be directed to the corresponding author at the email address listed on the title page of the manuscript, although any potential access remains subject to approval by the relevant collaborating parties and the terms of the underlying agreement. We appreciate the reviewer’s suggestion, which helped us improve the transparency of the revised manuscript regarding data access limitations.
